# Supplementary material for: Probing the Dynamics of Li+ Ions on the Crystal Surface: A Solid-State NMR Study
Source: Polymers (Basel). 2020 Feb 9;12(2):391. doi: 10.3390/polym12020391 (PMC7077695; doi:10.3390/polym12020391)
Supplement: Supplementary file 1 [file polymers-12-00391-s001.pdf]

polymers

MDPI

Supplementary Materials:

Probing the Dynamics of Li^+ Ions on the Crystal Surface: A Solid-State NMR Study

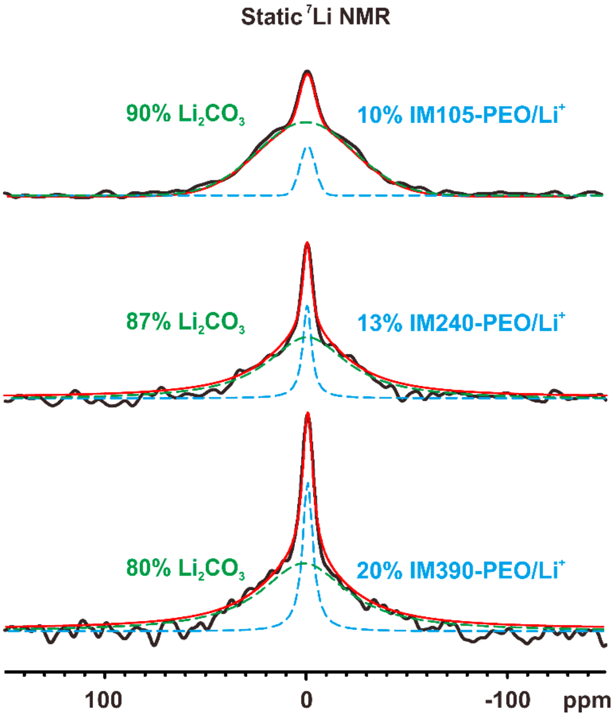

Figure S1. The static quantitative ^{7}Li NMR spectra of the mixture of IM-PEO/Li^+ and Li_2CO_3 powder with different immersion time (black lines). The sample contains 13 mg IM240-PEO/Li^+ and 8 mg Li_2CO_3. The experimental temperature is at 305 K. The blue dotted lines and green dotted lines denote the fitting peaks of IM-PEO/Li^+ and Li_2CO_3, respectively. The red lines denote the fitting peak of the mixture of IM-PEO/Li^+ and Li_2CO_3.

The ratio between Li^+ and oxygen atoms in the coordination structure was determined as the following (using IM240-PEO/Li^+ as the example): Firstly, we measured the quantitative ^{7}Li NMR spectrum on a mixture of Li_2CO_3 and IM240-PEO/Li^+ in which the weight of Li_2CO_3 and IM240-PEO/Li^+ were known. By comparing the signal of Li_2CO_3 and with that of IM240-PEO/Li^+, we could obtain the Li^+ concentration of IM240-PEO/Li^+ (mol/g). From DSC, we obtained the amorphous content of IM240-PEO/Li^+ from which the mole concentration of EO was obtained (mol/g). Combination of the ^{7}Li NMR the DSC measurement then yields the ratio of Li^+/EO in the samples.

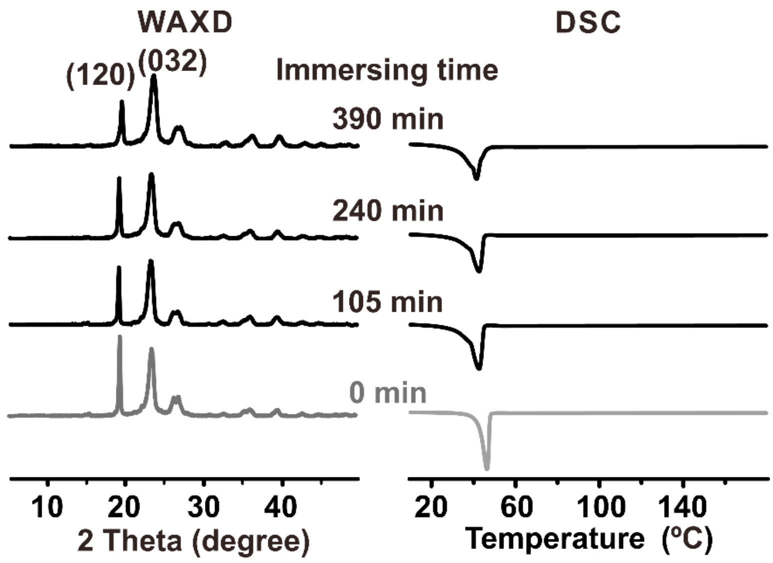

Figure S2. The WAXD patterns and DSC curves of the samples prepared using the different immersing time. The WAXD patterns were acquired at room temperature.

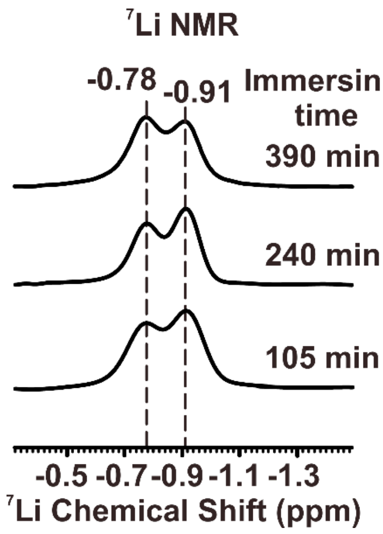

Figure S3. The ^{7}Li NMR spectra of the samples prepared using the different immersing time. The experimental temperature was 300 K.

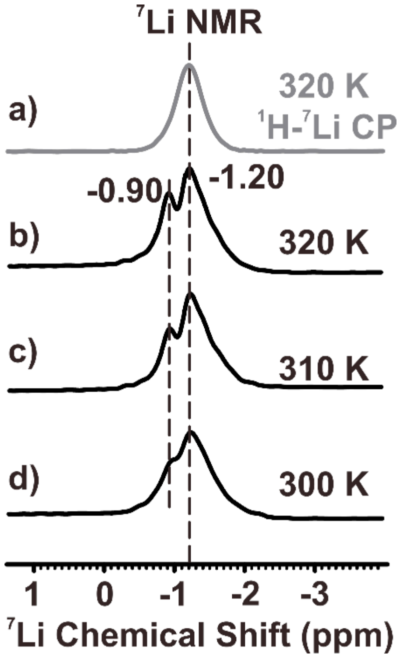

Figure S4. The ^{7}Li NMR spectra of the $(PEO)_3LiCF_3SO_3$. **a**): ^{7}Li CP/MAS spectrum with a ^{1}H-^{7}Li contact time of 500 μs. **b**) - **d**): The ^{7}Li single-pulse NMR spectra, acquired at different temperatures.

Figure S4a and S4b show the ^{1}H-^{7}Li CP/MAS and ^{7}Li single pulse spectra of $(PEO)_3LiCF_3SO_3$. In the ^{1}H-^{7}Li CP/MAS spectrum, only a broad peak centered at −1.2 ppm appears. This indicates that the Li^+ ions associated to this signal have a relatively strong ^{1}H-^{7}Li dipole coupling, which is typical in the crystalline PEO/Li^+ complexes. The disappearance of the ^{7}Li signal centered at −0.91 ppm can be attributed to the weak ^{1}H-^{7}Li dipole coupling, which is often observed in the amorphous regions of the PEO/Li^+ complexes. In the ^{7}Li single pulse spectrum of $(PEO)_3LiCF_3SO_3$ in Figure S4b, both the signals at −0.90 ppm and −1.20 ppm are observed. This is because the single pulse sequence cannot differentiate the amorphous and crystalline signals in the given experimental condition. Figure S4c and S4d show the ^{7}Li single pulse spectra of $(PEO)_3LiCF_3SO_3$, acquired at 310 K and 300 K. It is observed that with increasing temperature the signals at −0.90 ppm become clearer and stronger, whereas the signal at −1.20 ppm remains almost unchanged. This is well in line with our signal assignment that the signal at −0.90 ppm is the amorphous signal and the signal at −1.20 ppm is the crystalline signal.

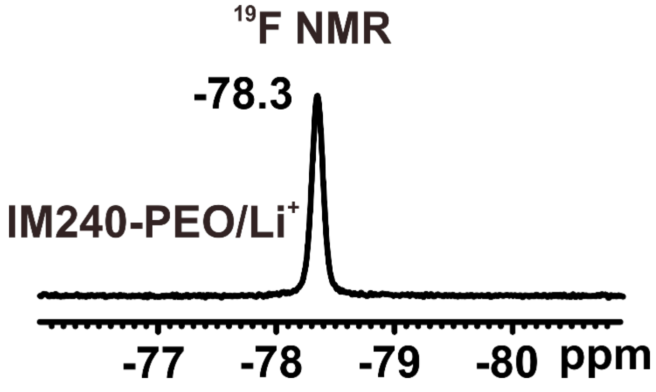

Figure S5. The ^{19}F single pulse MAS NMR spectrum of IM240-PEO/Li^+. The experimental temperature is 300 K.

The state of anions in the IM240-PEO/Li^+ sample is an interesting question, but not clear at this point. To have an electrostatic equilibrium, the anions must also get into the crystal surface regions together with the Li^+ ions. Meanwhile, although much larger than Li^+ ion, the anion $CF_3SO_3^-$ is relatively small compared with the interstices between the PEO chains in the amorphous regions. Therefore, the anion $CF_3SO_3^-$ will not only cover the surface of the lamellar segments. According to the literatures *(Macromolecules 1999, 32, 808-813.; Science 1993, 5135, 883-885.)*, Li^+ ions can form a stale coordination structure consisting of three ether oxygen atoms and one oxygen from each of two different $CF_3SO_3^-$ anions. We believe that similar coordination structures will also exist on the crystal surface regions. Figure S5 shows the ^{19}F NMR spectrum of IM240-PEO/Li^+. Only one signal observed in the spectrum, indicating that the anions only have one state in the sample.
